# Oscillatory Activity in the Cortex, Motor Thalamus and Nucleus Reticularis Thalami in Acute TTX and Chronic 6-OHDA Dopamine-Depleted Animals

**DOI:** 10.3389/fneur.2018.00663

**Published:** 2018-08-28

**Authors:** Laura C. Grandi, Alain Kaelin-Lang, Gergely Orban, Wei Song, Agnese Salvadè, Alessandro Stefani, Giuseppe Di Giovanni, Salvatore Galati

**Affiliations:** ^1^Laboratory for Biomedical Neurosciences, Neurocenter of Southern Switzerland, Taverne, Switzerland; ^2^Department of Neurology, Inselspital, Bern University Hospital, University of Bern, Bern, Switzerland; ^3^Faculty of Biomedical Sciences, Università della Svizzera Italiana, Lugano, Switzerland; ^4^Department System Medicine, UOSD Parkinson, University of Rome Tor Vergata, Rome, Italy; ^5^Department of Physiology and Biochemistry, Faculty of Medicine and Surgery, University of Malta, Msida, Malta; ^6^Neuroscience Division, School of Biosciences, Cardiff University, Cardiff, United Kingdom

**Keywords:** motor thalamus, nucleus reticularis thalami, 6-hydroxydopamine, tetrodotoxin, Parkinson's disease, neuronal oscillations

## Abstract

The motor thalamus (MTh) and the nucleus reticularis thalami (NRT) have been largely neglected in Parkinson's disease (PD) research, despite their key role as interface between basal ganglia (BG) and cortex (Cx). In the present study, we investigated the oscillatory activity within the Cx, MTh, and NRT, in normal and different dopamine (DA)-deficient states. We performed our experiments in both acute and chronic DA-denervated rats by injecting into the medial forebrain bundle (MFB) tetrodotoxin (TTX) or 6-hydroxydopamine (6-OHDA), respectively. Interestingly, almost all the electroencephalogram (EEG) frequency bands changed in acute and/or chronic DA depletion, suggesting alteration of all oscillatory activities and not of a specific band. Overall, δ (2–4 Hz) and θ (4–8 Hz) band decreased in NRT and Cx in acute and chronic state, whilst, α (8–13 Hz) band decreased in acute and chronic states in the MTh and NRT but not in the Cx. The β (13–40 Hz) and γ (60–90 Hz) bands were enhanced in the Cx. In the NRT the β bands decreased, except for high-β (Hβ, 25–30 Hz) that increased in acute state. In the MTh, Lβ and Hβ decreased in acute DA depletion state and γ decreased in both TTX and 6-OHDA-treated animals. These results confirm that abnormal cortical β band are present in the established DA deficiency and it might be considered a hallmark of PD. The abnormal oscillatory activity in frequency interval of other bands, in particular the dampening of low frequencies in thalamic stations, in both states of DA depletion might also underlie PD motor and non-motor symptoms. Our data highlighted the effects of acute depletion of DA and the strict interplay in the oscillatory activity between the MTh and NRT in both acute and chronic stage of DA depletion. Moreover, our findings emphasize early alterations in the NRT, a crucial station for thalamic information processing.

## Introduction

Recent evidence has suggested that abnormal oscillatory activity at specific frequencies within basal ganglia (BG) and cortex (Cx) represents a hallmark of Parkinson's disease (PD) (1–10). This abnormal oscillatory activity may reflect dysfunctions of cortico-BG-thalamo-cortical loop linked to Parkinsonian symptoms in both PD patients and/or animal models of this disease (8–11) and its recognition could provide possible biomarkers for the disease state.

Brain oscillatory activities are classically segmented into different frequency band intervals, i.e., 2–4 Hz (delta, δ), 4–8 Hz (theta, θ), 8–13 Hz (alpha, α), 13–40 Hz (beta, β), and 60–90 Hz (gamma, γ). Each band is associated with one or more specific physiological behavior and differently contribute to information processing (12). β oscillations are involved in motor control and are greatly enhanced at different sites within the BG circuit in both PD patients and animal models of PD (9, 11, 13, 14). In addition, elevated β band synchronization could be considered as an expression of bradykinesia (13, 15). As proof of its strong association with motor signs in PD, β activity is reduced by dopaminergic therapies (15, 16). Recent evidence (17) supports the idea of functional subdivision of this band in low-β (Lβ, 15–20 Hz) and high-β (Hβ, 25–30 Hz). Lβ in the subthalamic nucleus (STN) is tightly associated with Parkinsonian symptoms in patients that do not receive medications, whereas Hβ reflects the degree of coupling between cortical and STN activity (18, 19). Nevertheless, the exact role of Lβ and Hβ band in PD remains an unsolved question.

Opposite to β, γ band is supposed to be associated to dyskinesia (20) and more generally to modulation of movements (20). In particular, γ band is involved in voluntary movements (21, 22), but also in motor imagery (23), as well as in planning of movements (24).

In addition, γ band has also been related to sensory and cognitive processing (25), attention, long-term memory and language tasks (26, 27). In PD patients, a γ decrease has been shown during anti-Parkinsonian therapies (15, 28). In line, deep brain stimulation (DBS) of STN at γ frequencies facilitates movements (29) and it is powerfully expressed in both Cx and globus pallidus (GP) in levodopa-induced dyskinesia (LID) in 6-hydroxydopamine (6-OHDA)-lesioned rats (20, 30). Concerning STN DBS, a correlation between frequency of stimulation and improvement of symptoms has been recently shown (31). For example, during 5 Hz DBS, a worsening of bradykinesia has been shown, while both bradykinesia and tremor showed no improvement at frequencies below 50 Hz (32).

The θ band has been described in frontal and central cortical regions (33), as implicated in several functions different from the control of voluntary movements, such as sensory processing and memory in healthy people (34). The θ band increased in PD patients, selectively during a motor task (35), as well as in PD patients experiencing freezing of gait (36).

The δ band is instead associated with sleep functions (37) as well as with cognitive processes (38).

It has been shown the association between δ band disruption with PD (39–41). For instance, the administration of the Delta Sleep-inducing peptide into the SNc induces Parkinsonian syndrome in rat (42).

Moreover, Parker and colleagues (43) showed that δ expression on medial frontal cortex (MFC) is associated with cognitive dysfunctions in both PD patients and animal models and DA depletion in the MFC. In addition, sleep disturbances are common symptoms in PD (44) and often they arise before the onset of motor symptoms (45). Although, the DAergic treatment seems have no effect of sleep functions (41, 46), some might have positive effects on sleep quality (47).

Finally, α frequency, according to the inhibition-timing hypothesis (48), is negatively correlated with cortical excitability and its enhancement prevents task-irrelevant interference (49). The thalamic- and cortical-generated α activity has a role in attentive tasks in physiological conditions (50–52) and it is modulated by visual task performance in occipital lobe (53). In addition, α oscillation is modulated by visual stimuli (54), even if they are sub- and supraliminal stimuli (55). In line, correlation between the phase of α oscillatory activity and the saccadic reaction time in cognitive task responses has been reported (56). It has been hypothesized that changes in α band expression might underlie some cognitive and attentive difficulties observed in PD patients (57). Within the BG circuit, the sensory-motor thalamus (MTh) has critical role in motor information processing (58), but contrasting data exist concerning its neuronal activity in PD (59). According to the *searchlight hypothesis*, the nucleus reticularis thalami (NRT) has a fundamental role as the guardian of the thalamus, contributing to the encoding of thalamic information (60–68). In particular, the sensorimotor MTh is modulated by the NRT motor sector.

In spite of its importance, the oscillatory activity across multiple frequency bands within the MTh and the NRT is a neglected area in PD studies. Therefore, we monitored the electrocorticogram (ECoG) and the local field potentials (LFPs) of the MTh and the NRT in two dopamine (DA) depletion states in a PD animal model. We first performed our recordings in the standardized Parkinsonian animal model obtained with the injection of 6-OHDA, capable of causing a chronic DA depletion.

Additionally, since it has been shown that some PD symptoms, such as bradykinesia, are already associated with acute DA depletion state induced by tetrodotoxin (TTX), we performed a similar study in animal with acute DA depletion induced by TTX (69–74).

We hypothesized that the oscillatory activity within MTh-NRT might be different in acute DA depletion state from that recorded in chronic 6-OHDA-lesioned rats, due to the presence of adaptive mechanisms.

## Methods

### Ethical approval

All experimental electrophysiological and histological procedures were carried out in compliance with Switzerland laws on animal experimentation and approved by the Animal Research Committee and the Veterinary Office of the Canton of Ticino, Switzerland (TI-08-2015). We analyzed 42 adult male Sprague Dawley rats weighing ~300 g.

### Pre-recording surgery

Rats were anesthetized with urethane (1.4 g/kg, i.p.) (Sigma Chemical Co., St Louis, MO, USA) and mounted on a stereotaxic instrument (Stoelting Co., Wheat Lane, Wood Dale, IL, USA), maintaining the body temperature at 37–38°C with a heating pad (Stoelting Co., Wheat Lane, Wood Dale, IL, USA) placed beneath the animal. A midline scalp incision was made, the skull was drilled on the left side and the dura was then spread out to expose the cortical surface. All wound margins were infiltrated with a local anesthetic (lidocaine 0.5%). All electrophysiological recordings were performed in three categories of animals: in normal rats, in 6-OHDA-lesioned rats and in acutely DA-depleted animals (see Table 1).

**Table 1:** Animals utilized in the study.

**Animal groups for electrophysiology**	**Sacrificed**	**Analyzed**
CTL rats	10	6
TTX-treated rats	34	27
6-OHDA-lesioned rats	15	9
Total	59	42

*CTL, control; TTX, tetrodotoxin; 6-OHDA, 6-hydroxydopamine*.

### Unilateral 6-OHDA lesioning

Chronic DA depletion was induced by performing a unilateral 6-OHDA denervation in the left hemisphere with standard technique (75, 76). The animals were anesthetized with 1.5–2.5% isoflurane in oxygen, mounted on a stereotaxic instrument (Stoelting Co., Wheat Lane, Wood Dale, IL, USA) for the injection of the neurotoxin (8 μg/4 μl of saline solution containing 0.1% of ascorbic acid) in the medial forebrain bundle (MFB; stereotaxic coordinates: 2.56 mm posterior to the bregma, 2 mm lateral to the midline, and 8.6 mm below the cortical surface). The electrophysiological recordings were performed 21–29 days after the surgery.

### Pharmacological blockade of the medial forebrain bundle

The pharmacological blockade of the MFB was performed according to previous publications (55–57). TTX was injected via inverse microdialysis by using a probe with 1 mm dialytic membrane (CMA/11 microdialysis probe, CMA Microdialysis, Stockholm, Sweden). TTX was perfused by using a syringe pump (CMA/400, CMA Microdialysis, Stockholm, Sweden) with a rate flow of 1 μl/min, for 10 min.

### Electrophysiological recordings

The ECoG was recorded through a screw electrode (Dentorama, Stockholm, Sweden, 8 mm of total length, 3 mm tip lenght) placed on the cortical surface above the right frontal Cx (3.0 mm anterior of bregma and 2.0 mm lateral to the midline) and referenced against an indifferent screw electrode placed above cerebellum. Raw ECoG was band-pass filtered (0.1–300 Hz) and amplified (× 2000; Neurolog). The ECoG was on-line digitalized with a sample rate of 600 Hz through an analogical/digital interface (Micro1401 mk II, Cambridge Electronic Design, Cambridge, UK) and stored on a computer for the subsequent inspection. During cortical recordings, we collected LFPs from the left MTh or the NRT (from 1.2 mm to 1.8 posterior of bregma and from 2 to 2.6 mm lateral to the midline). The recordings were performed using tungsten electrodes (Word Precision Instrument, USA, TM33B01). At the end of the recordings, the animals were sacrificed. The recordings were carried out 21–29 days after the administration of 6-OHDA, while in TTX-treated animals, after TTX infusion.

### LFP and ECoG analysis

**Figure 1:**
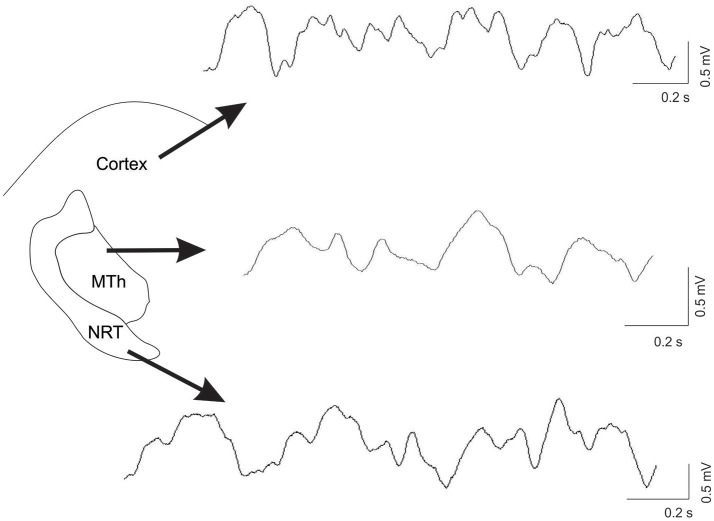
Example of recordings, from the ECoG trace, the MTh and the NRT in control condition. The raw data was processing with smoothing.

The local field potentials were analyzed by Spike2 script (SUDSA22) to calculate the total power of δ band (δ, 2–4 Hz), θ band (θ, 4–8 Hz), α band (α, 8–13 Hz), low-β band (Lβ, 13–25 Hz), high-β band (Hβ, 25–40 Hz), and γ band (γ, 60–90 Hz) in the Cx, MTh, and NRT of control (CTL), acute and chronic DA-depleted rats. The analysis was performed with raw data in the first 6 min of recording using the fast Fourier transform (FFT) analysis (4096 points). Figure 1 represents an example of recording, with smoothing signal.

### Statistical analysis

For the comparison of total power of analyzed bands, among CTL vs. TTX and 6-OHDA we performed the non-parametric Kruskal Wallis test followed by Mann Whitney U test for the comparisons CTL vs. TTX, CTL vs. 6-OHDA, and TTX vs. 6-OHDA. For each statistical analysis a value of *p* < 0.05, corrected per number of comparisons (*n* = 3), therefore *p* = 0.016, was considered statistically significant. For each condition, we calculated the mean of each of the 6 min and then compared the 6 min among conditions in the MTh and the NRT. The cortical bands were calculated as ECoG recorded during MTh and during NRT neurons. Therefore, the comparisons were made on the mean of 6 min (*n* = 6) for each structures. The results are expressed as mean ± SEM. For exact *p* value, please refer to Results section.

The ECoG and the LFP from the MTh and NRT was divided into the six different frequency bands. The total power of each band was calculated and compared among CTL, acute, and chronic DA depletion states (Supplementary Tables 1–3). Then the percentage of change in comparison to CTL was calculated for each band in the Cx, the MTh and the NRT (Supplementary Tables 4A–C).

We recorded from a total of 59 rats (CTL *n* = 10, TTX-treated *n* = 34, and 6-OHDA-denervated rats *n* = 15) and analyzed from a total of 42 animals (CTL *n* = 6; TTX-treated *n* = 27 and 6-OHDA-denervated rats *n* = 9). In detail, in CTL rats we analyzed a total of 7 LFP recordings from the NRT and 15 LFP recordings from the MTh, recorded parallel to EEG (total of *n* = 22); in TTX-treated rats we analyzed 11 LFP recordings from the NRT and 16 LFP recordings from the MTh, recorded parallel to EEG (total of *n* = 27); in 6-OHDA rats we analyzed 5 LFP recordings from the NRT and 15 LFP recordings from the MTh, recorded parallel to EEG (*n* = 20).

## Results

Overall, after DA depletion the magnitude of changes of oscillatory activity in all analyzed frequency ranges within the NRT was more marked than that within the MTh and the Cx. Figure 1 shows an example of recordings in control condition in the Cx, the MTh and the NRT.

### Effects of DA-depletion on cortical oscillatory activities

The cortical activity changed after both chronic and acute DA depletion, with exception of α band (CTL: 0.0065 ± 0.0004; acute state: 0.0069 ± 0.00047; chronic state: 0.0064 ± 0.0003).

The δ band decreased of 20.5% in chronic DA depletion state (δ: 0.0439 ± 0.0009 in CTL, 0.026 ± 0.0052 in acute state and 0.0349 ± 0.0011 in chronic state; CTL vs. acute state *p* = 0.021, CTL vs. chronic state *p* = 0.000, acute vs. chronic state *p* = 1).

The θ band decreased in acute (32.3%) and chronic (17.2%) DA depletion states (θ: 0.0254 ± 0.0005 in CTL, 0.017 ± 0.002 in acute state, and 0.021 ± 0.0006 in chronic state; CTL vs. acute state *p* = 0.000, CTL vs. chronic state *p* = 0.000, acute vs. chronic state *p* = 0.299).

**Figure 2:**
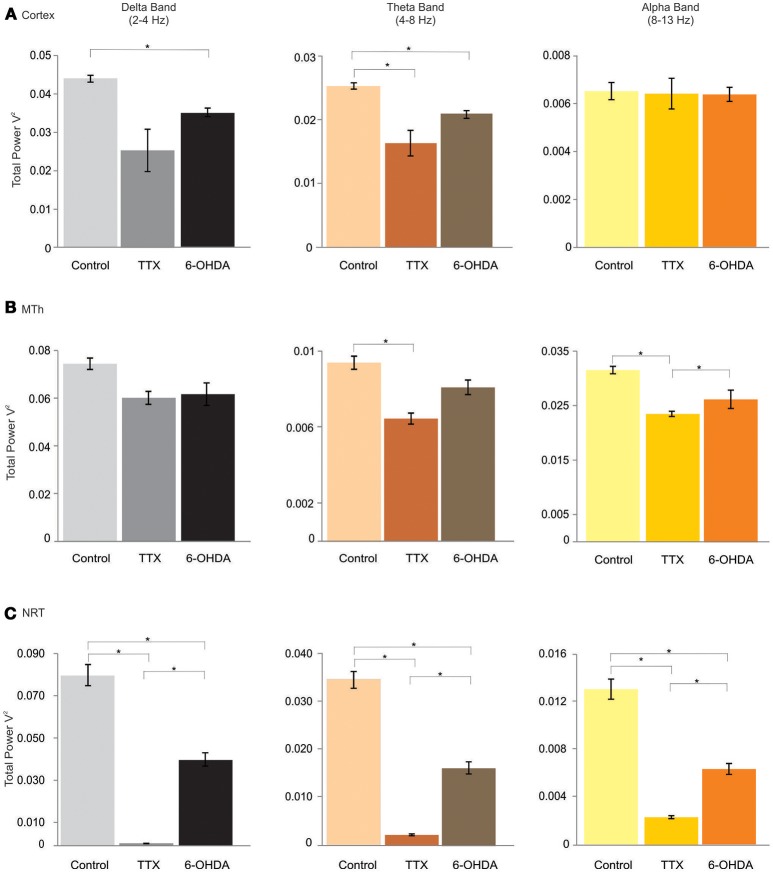
δ (delta), θ (theta), and α (alpha) bands of the cortex (Cx), the MTh and the NRT (from top), in control, acute (TTX-infused rats) and chronic (6-OHDA-denervated rats) DA-depletion states. ^*^p < 0.016, Mann Whitney Test.

**Figure 3:**
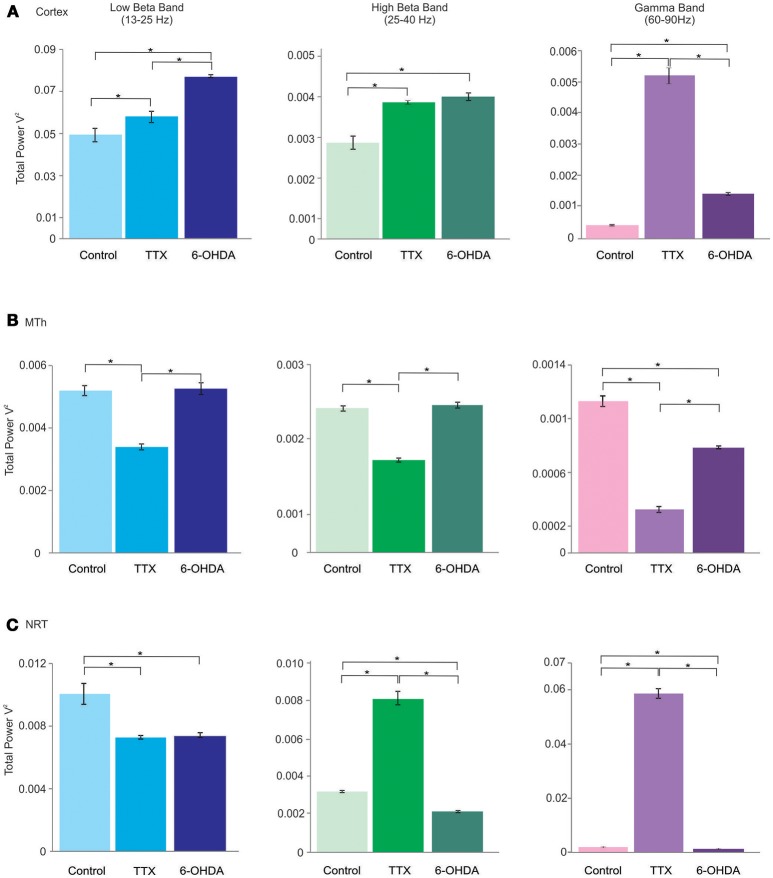
Lβ (low beta), Hβ (high beta) and γ (gamma) bands of the cortex (Cx), the MTh and the NRT (from top), in control, acute (TTX-infused rats) and chronic (6-OHDA-denervated rats) DA-depletion states. ^*^p < 0.016, Mann Whitney test.

The Lβ, Hβ, and γ band frequencies increased in both acute and chronic DA depletion state (Lβ: 0.0049 ± 0.0003 in CTL, 0.0066 ± 0.0001 in acute state and 0.0077 ± 0.00007 in chronic state; CTL vs. acute state *p* = 0.000, CTL vs. chronic state *p* = 0.000. acute vs. chronic state *p* = 0.000. Hβ: 0.0024 ± 0.0002 in CTL, 0.0038 ± 0.00018 in acute state and 0.00398 ± 0.00009 in chronic state; CTL vs. acute state *p* = 0.000, CTL vs. chronic state *p* = 0.000, acute vs. chronic state *p* = 0.686. γ: 0.0004 ± 0.00002 in CTL, 0.0052 ± 0.00024 in acute state and 0.0014 ± 0.00004 in chronic state; CTL vs. acute state *p* = 0.000, CTL vs. chronic state *p* = 0.000, acute vs. chronic state *p* = 0.000). The Lβ increased by 34.9 and 55.6% in acute and chronic DA depletion state, respectively, the Hβ increased by 62% in acute state and of 67.9% in chronic DA depletion state, whilst the γ band increased by 1258.4% and of 261.6% in chronic state, in acute and chronic depletion states, respectively (Figures 2A, 3A; Supplementary Tables 1A,B, 4A).

These results underlid that the cortical oscillatory activity in low frequencies range seems to be negatively affected by DA-depletion states, with exception of α band, that instead did not change in any conditions. On the contrary, the DA-depletion states tend to increase the oscillations in high frequencies ranges (Lβ, Hβ, and γ band). In addition, the results show that the cortical activity seems to be affected not just in chronic DAergic denervation but also in acute state, induced by TTX.

### Effects of DA-depletion on MTh oscillatory activities

In the MTh (Supplementary Tables 2A,B; Figures 2B, 3B), the acute and chronic DA depletion affected differently the oscillatory activity. In particular, the δ (0.0745 ± 0.0024 in CTL, 0.0601 ± 0.0027 in acute state and 0.062 ± 0.0047 in chronic state) did not change in DA depletion states in comparison to CTL. The θ and α bands decreased in acute state (θ: 0.0315 ± 0.0007 in CTL, 0.0235 ± 0.0005 in acute state and 0.0262 ± 0.0017 in chronic state; CTL vs. acute state *p* = 0.004, CTL vs. chronic state *p* = 0.037, acute vs. chronic state *p* = 0.337. α: 0.0094 ± 0.0003 in CTL, 0.0064 ± 0.0003 in acute state and 0.0081 ± 0.0004 in chronic state; CTL vs. acute state *p* = 0.004, CTL vs. chronic state *p* = 0.025, acute vs. chronic state *p* = 0.01). In particular, θ band decreased of 25.5% in acute state, whilst α band decreased of 31.7% in acute state (Supplementary Table 4B). Similarly, the Lβ and Hβ bands decreased just in acute DA depletion of 34.6 and 35.7%, respectively (Supplementary Table 4B), respectively (Lβ: 0.0052 ± 0.0002 in CTL, 0.0034 ± 0.00009 in acute state and 0.0053 ± 0.0002 in chronic state; CTL vs. acute state *p* = 0.004, CTL vs. chronic state *p* = 1, acute vs. chronic state *p* = 0.004. Hβ: 0.0019 ± 0.00004 in CTL, 0.0012 ± 0.00003 in acute state and 0.0019 ± 0.00004 in chronic state, CTL vs. acute state *p* = 0.004, CTL vs. chronic state *p* = 0.262, acute vs. chronic state *p* = 0.004).

The γB decreased in both acute and chronic DA depletion state of 71.8 and 30.8%, respectively (0.0011 ± 0.00004 in CTL, 0.0003 ± 0.00002 in acute state and 0.0008 ± 0.00001 in chronic state; CTL vs. acute state *p* = 0.004, CTL vs. chronic state *p* = 0.004, acute vs. chronic state *p* = 0.004).

Interestingly, these results show that the MTh oscillatory activity is strongly and mainly affected by acute DA depletion state. Indeed, TTX, but not 6-OHDA, with exception of δ and γ bands, induced the decrease of all analyzed bands.

### Effects of DA-depletion on the NRT oscillatory activities

In the NRT (Supplementary Tables 3A,B; Figures 2C, 3C), the acute and chronic DA depletion states changed all the analyzed bands. In particular, δ (0.0806 ± 0.005 in CTL, 0.0010 ± 0.00007 in acute state and 0.0408 ± 0.0032 in chronic state, CTL vs. acute state *p* = 0.004, CTL vs. chronic state *p* = 0.004, acute vs. chronic state *p* = 0.004), θ (0.0347 ± 0.0018 in CTL, 0.002 ± 0.0002 in acute state and 0.0163 ± 0.0013 in chronic state, CTL vs. acute state *p* = 0.004, CTL vs. chronic state *p* = 0.004, acute vs. chronic state *p* = 0.004), α (0.013 ± 0.0008 in CTL, 0.002 ± 0.0001 in acute state and 0.0063 ± 0.0005 in chronic state, CTL vs. acute state *p* = 0.004, CTL vs. chronic state *p* = 0.004, acute vs. chronic state *p* = 0.004) and Lβ (0.0101 ± 0.0007 in CTL, 0.0073 ± 0.0001 in acute state and 0.0074 ± 0.0001 in chronic state, CTL vs. acute state *p* = 0.004, CTL vs. chronic state *p* = 0.004, acute vs. chronic state *p* = 0.522) bands decreased in both acute and chronic DA depletion state. Hβ (0.0032 ± 0.00005 in CTL, 0.0083 ± 0.0003 in acute state and 0.0022 ± 0.000043 in chronic state, CTL vs. acute state *p* = 0.004, CTL vs. chronic state *p* = 0.004, acute vs. chronic state *p* = 0.004) and γ (0.0014 ± 0.00003 in CTL, 0.059 ± 0.0017 in acute state and 0.00097 ± 0.00003 in chronic state, CTL vs. acute state *p* = 0.004, CTL vs. chronic state *p* = 0.004, acute vs. chronic state *p* = 0.004) increased in acute state and decreased in chronic state.

The δ, θ, α, and Lβ bands decreased by 98.8, 94.3, 83.4, and 27.8% in acute state, respectively, and by 49.4, 53, 51.4, and 27.2% in chronic state. The Hβ and γ bands increased by 154.8 and 4055.1% in acute state and decreased by 32.1 and 31.6% in chronic state (Supplementary Table 4C).

The results show that the NRT is strongly affected by both acute and chronic DA depletion states, differently from MTh. Indeed, the δ, θ, α, and Lβ bands, decreased in both DA depletion states. Interestingly, in the high frequencies range (Hβ and γ bands), the activity increased in acute DA depletion state and instead decreased in chronic state.

## Discussion

Compelling evidence shows that abnormal oscillatory activity within the Cx and BG circuit mainly in the β range, but not only, contributes to motor impairments in PD (13). On the other hand, the effects of DA depletion in crucial structures of the cortico-subcortical loop such as the MTh and its principal modulator, i.e., the NRT have been poorly investigated. In order to shed more light on this important field, we investigated band oscillations in the cortical and subcortical MTh-NRT loop in rats in both acute and chronic DA-depleted states.

### Cortical and NRT δ band is affected by acute and chronic DA depletion

In line with the observation of a δ decrease in cognitively normal PD patients (39), we found a reduction of δ wave power at cortical level in chronic DA-depleted state. In addition, we found that δ wave also decrease after early acute DA-depletion NRT. The δ band is associated with sleep modulation and disruption of this activity reflect sleep-disorders (77). Interestingly, one of the most common symptom in early stage of PD concerns sleep deficits (44). In addition, frequencies around δ power intervals are associated with PD tremor and are detected in the STN in decision conflict situations (78). Our results showed a decrement of oscillations in δ frequency in both thalamic nuclei and Cx.

### Cortical and thalamic θ band decreases in both acute and chronic depletion state

Contrary to previous reports (79, 80), we observed a decrease of θ activity in both acute and chronic DA-depleted states in the three investigated areas. In the NRT we found an increase in chronic DA depletion state in comparison to acute state, without nevertheless reach the baseline level. Cavanagh and colleagues demonstrated that in PD patients the θ power in the MPC and the STN is associated with decision conflict situations and that STN-DBS alters this coupling (78). Therefore, θ power increases in frontal Cx, associated with PD in a specific task conditions, while it decreases in our anesthetized PD animal model.

### Thalamic α band decreases in acute and chronic DA depletion states

According to previous report, decrease of cortical α power correlates with dementia (57). Whilst we failed to find any changes in cortical α band, it decreased in the MTh and the NRT. In particular, NRT-α power decreased in both acute and chronic DA depletion in comparison to control, whereas in the MTh it decreased just in acute state. The power of α frequency was higher in chronic than acute DA depletion conditions. Consistently, the thalamus is supposed to be the α band rhythms generator (79), as postulated by the inhibition-timing hypothesis of α oscillations (48, 81).

### Cortical β bands increase in DA depletion state, whilst it decreases in MTh in acute state

The β band is one of the most studied oscillatory activity critically involved in PD (8, 11, 13, 82). In physiological conditions it is suppressed by motions (83), whilst its impairment leads to deficits in complex sensorimotor processes such as repetitive movements (84, 85) and it is pathophysiological relevant to bradykinesia (10, 15). More precisely, it has been reported a correlation between rigidity and bradykinesia and the β band (86). Moreover, Lβ band (12–30 Hz) shows a decrease in power in response to dopaminergic treatment (87). Hβ power in STN is enhanced in patients with freezing of gait in comparison to patients without this common PD characteristic (88). In addition, the Lβ band is prominent in inattentive state, whilst it has been observed a shift to Hβ band during walking in the substantia nigra pars reticulata (SNr) of chronically 6-OHDA-denervated rats (17).

Here, we found an increment of cortical Lβ and Hβ band in both acute and chronic DA depletion state. Compared to the Cx, the thalamic β activity is differently affected by DA depletion. In MTh, β band power is decreased in acute state. In NRT, the Lβ band is decreased in both acute and chronic state, whilst the Hβ band is increased in acute state.

In addition, our results support the idea that β band has cortical and not thalamic origins (89). In particular, we observed that the cortical β band increased in both acute and chronic DA depletion states whilst MTh and NRT bands are differently modulated. The MTh Lβ and Hβ decreased just in acute state, whilst NRT Hβ increased in acute state and decreased in chronic state. The NRT Lβ decreased instead in both acute and chronic DA depletion states. Interestingly, in chronic state the β band in the MTh did not change in comparison to control and this may be due to the fact the MTh is affected by opposite influence by the Cx and the NRT.

### γ band is affected by both acute and chronic DA depletion

As it has been previously reported (90, 91), TTX-treated and 6-OHDA-lesioned rats showed an increment of the oscillatory activity in the Cx in the γ frequency. Similarly, NRT activity increased, whilst MTh γ activity is decreased. This increase of cortical and NRT γ band could be considered as a basis for developing of dyskinesia during levo-dihydroxyphenylalanine (L-DOPA) treatment. The cortical γ activity is coupled with thalamic α oscillations (92). We found that cortical γ and thalamic α bands showed opposite behaviors, indeed the DA depletion states determined the increment of cortical γ power and decrement of the thalamic α band.

## Conclusion

Taking together, the evidence from literature and the present results reveal an evident complex oscillatory pattern of neuronal activity in PD, at the level of different nuclei of BG-thalamic-cortical network. Furthering our understanding of these aberrant oscillations will likely contribute to the advance of early diagnosis based on non-invasive investigation of brain activity.

Our results support the idea that there is not a unique band responsible of the PD pathological mechanisms, instead all bands could contribute to the pathological complexity of the oscillatory activity. Importantly, since the chronic DA depletion state did not drastically affect the thalamic oscillatory activity, our data raise the possibility that some aspects of these oscillatory activity in PD may be promoted by the acute DA loss (69, 70, 93), and the involvement of the NRT. The injection of TTX in MFB is accompanied by increase of cortical β and γ bands, as typically recorded in chronic DA denervation and in PD patients (13, 15, 20). The MTh oscillations change occurs preferentially in acute DA depletion state, while not in chronic state due to the fact that it may be compensated by the NRT activity. In the acute DA depletion state, the changes in different BG circuit sites, such as SN and GP (69, 70, 93) might instead result in the observed changes of thalamic activity.

This result could be considered an important starting point in order to shed some light on the role of the NRT, a structure usually neglected in PD pathophysiology, in a hypothetical widely Cx-BG network. Therefore, the thalamic information is processed in the NRT, and may enhance or suppress thalamic responsiveness, depending on the relative timing of afferent inputs and NRT activation (94). The NRT is implicated in a variety of functions, such as motor, arousal, sleep modulation, sensory, and associative stimuli coding (95), and each NRT sector encodes the relative specific information. Nevertheless, since it is a small and deep brain structure, it is difficult to investigate it *in vivo*, and elucidate its specific role in modulating larger-scale brain activity. Early models of the NRT functions posit that thalamocortical and NRT neurons are reciprocally innervated (96), determining the oscillatory phenomena (97, 98). However, computational models support the idea that an open-loop could explain the thalamic-NRT circuit. Accordingly, low-threshold bursting in an open-loop circuit could be consider a mechanism by which the NRT may paradoxically enhance thalamocortical activation, depending on the relative timing of the NRT and thalamocortical neurons (95). This dynamic NRT-thalamic-cortical loop could explain the hypothetical role of the NRT for thalamocortical modulation (95).

In pathological conditions, the strong changes of the NRT oscillatory activities in acute DA depletion state could explain the absence of acute cortical and the thalamic change and the later cortical and thalamic changes in chronic DA deficiency condition.

Our results are in line with the evidence of a strong influence of the NRT in cortical and thalamic firing mode modulation in physiological and pathological conditions involving dysfunctions of acetylcholine, nicotine and DA systems (99). Overall, the strong impairments of the NRT oscillatory activity in all analyzed frequencies in both acute and chronic DA depletion states may suggest a possible critical role of the NRT in both PD motor and non-motor symptoms, in early and late stages.

Our study has some caveats. Firstly, we have to consider that findings in PD animal models cannot totally be translated to human disease state. Moreover, we have to consider that (i) the dopaminergic depletion is not the unique feature of PD; (ii) the 6-OHDA lesion does not reflect totally the PD symptoms, and (iii) the electrophysiological recordings were performed under urethane anesthesia, rending impossible to explore if oscillatory activity depends on the motions and/or cognitive tasks, impaired in PD. In spite of these limitations, we think that our results represent an important starting point in order to better understand the changes of thalamo-cortical oscillations induced by dopaminergic denervation in PD.

## Author contributions

SG, GD, AlS, and AK-L conception and design. AgS, AlS, LG, GD, GO, and WS acquisition of data analysis and interpretation of data. AlS, LG, GD, and SG drafting the article and revising it.

### Conflict of interest statement

The authors declare that the research was conducted in the absence of any commercial or financial relationships that could be construed as a potential conflict of interest.
