# Supplementary material for: Oscillatory Activity in the Cortex, Motor Thalamus and Nucleus Reticularis Thalami in Acute TTX and Chronic 6-OHDA Dopamine-Depleted Animals
Source: Front Neurol. 2018 Aug 28;9:663. doi: 10.3389/fneur.2018.00663 (PMC6122290; doi:10.3389/fneur.2018.00663)
Supplement: Supplementary file 1 [file Table_1.DOC]

**Supplementary Material**

**Oscillatory activity in the cortex, motor thalamus and thalamic reticular nucleus in acute TTX and chronic 6-OHDA dopamine-depleted animals**

Laura Clara Grandi^1^, Alain Kaelin-Lang^1,2^, Gergely Orban^1^, Wei Song^1^, Agnese Salvadè^1^, Alessandro Stefani^3^, Giuseppe Di Giovanni^4^, Salvatore Galati^1§^

§Correspondence

Salvatore Galati, MD, PhD

Laboratory for biomedical neurosciences, Neurocenter of Southern Switzerland

via ai Söi, 24

6897 Taverne-TI-Switzerland

Tel.: +41 91 811 6789, Fax: +41 8116915, salvatore.galati@eoc.ch

**Supplementary Tables**

Table 1A. Cortical δ, θ and α band mean ± SEM.

Table 1B. Lβ and Hβ and cortical γ band mean ± SEM.

Table 2A. δ, θ and α band mean ± SEM, in the motor thalamus (MTh)

Table 2B. Lβ and Hβ and γ band mean ± SEM, in the motor thalamus (MTh)

Table 3A. δ, θ and α band mean ± SEM, in the reticular thalamic nuclei (NRT)

Table 3B. Lβ and Hβ and γ band mean ± SEM, in the reticular thalamic nuclei (NRT)

Table 1A. Cortical δ, θ and α bands

| Cx | Delta Band  (2-4 Hz) | Theta Band  (4-8 Hz) | Alpha Band  (8-13 Hz) |
| --- | --- | --- | --- |
| CTL | 0.0439 ± 0.0009 | 0.0254 ± 0.0005 | 0.0065 ± 0.0004 |
| TTX | 0.026 ± 0.0052 | 0.017 ± 0.002* | 0.0069 ± 0.00047 |
| 6-OHDA | 0.0349 ± 0.0011* | 0.021 ± 0.0006* | 0.0064 ± 0.0003 |

Cx: Cortex; CTL, control; TTX: tetrodotoxin; 6-OHDA: 6-hydroxydopamine

* p < 0.016 vs Control ** p < 0.016 TTX vs 6-OHDA

Table 1B. Cortical Lβ and Hβ and γ bands

| Cx | Low Beta Band  (13-25 Hz) | High Beta Band  (25-40 Hz) | Gamma Band  (60-90 Hz) |
| --- | --- | --- | --- |
| CTL | 0.0049 ± 0.0003 | 0.0024 ± 0.0002 | 0.0004 ± 0.00002 |
| TTX | 0.0066 ± 0.0001* | 0.0038 ± 0.00018* | 0.0052 ± 0.00024* |
| 6-OHDA | 0.0077 ± 0.00007*;** | 0.00398 ± 0.00009* | 0.0014 ± 0.00004*;** |

Cx: Cortex; CTL, control; TTX: tetrodotoxin; 6-OHDA: 6-hydroxydopamine

*p < 0.016 vs Control **p < 0.016 TTX vs 6-OHDA

Table 2A. Motor thalamus (MTh) δ, θ and α bands

| MTh | Delta Band  (2-4 Hz) | Theta Band  (4-8 Hz) | Alpha Band  (8-13 Hz) |
| --- | --- | --- | --- |
| CTL | 0.0745 ± 0.0024 | 0.0315 ± 0.0007 | 0.0094 ± 0.0003 |
| TTX | 0.0601 ± 0.0027 | 0.0235 ± 0.0005* | 0.0064 ± 0.0003* |
| 6-OHDA | 0.062 ± 0.0047 | 0.0262 ± 0.0017 | 0.0081 ± 0.0004** |

CTL, control; MTh: motor thalamus; TTX: tetrodotoxin; 6-OHDA: 6-hydroxydopamine

*p < 0.016 vs Control **p < 0.016 TTX vs 6-OHDA

Table 2B. Motor thalamus (MTh) Lβ and Hβ and γ bands

| MTh | Low Beta Band  (13-25 Hz) | High Beta Band  (25-40 Hz) | Gamma Band  (60-90 Hz) |
| --- | --- | --- | --- |
| CTL | 0.0052 ± 0.0002 | 0.0019 ± 0.00004 | 0.0011 ± 0.00004 |
| TTX | 0.0034 ± 0.00009* | 0.0012 ± 0.00003* | 0.0003 ± 0.00002* |
| 6-OHDA | 0.0053 ± 0.0002** | 0.0019 ± 0.00004** | 0.0008 ± 0.00001*;** |

CTL, control; MTh: motor thalamus; TTX: tetrodotoxin; 6-OHDA: 6-hydroxydopamine

*p < 0.016 vs Control **p < 0.016 TTX vs 6-OHDA

Table 3A. Reticular thalami nuclei (NRT) δ, θ and α bands

| NRT | Delta Band  (2-4 Hz) | Theta Band  (4-8 Hz) | Alpha Band  (8-13 Hz) |
| --- | --- | --- | --- |
| CTL | 0.0806 ± 0.0050 | 0.0347 ± 0.0018 | 0.013 ± 0.0008 |
| TTX | 0.0010 ± 0.00007* | 0.002 ± 0.0002* | 0.002 ± 0.0001* |
| 6-OHDA | 0.0408 ± 0.0032*;** | 0.0163 ± 0.0013*;** | 0.0063 ± 0.0005*;** |

CTL, control; NRT: thalamic reticular nucleus; TTX: tetrodotoxin; 6-OHDA: 6-hydroxydopamine

*p < 0.016 vs Control **p < 0.016 TTX vs 6-OHDA

Table 3B. Reticular thalami nuclei (NRT) Lβ and Hβ and γ bands

| NRT | Low Beta Band  (13-25 Hz) | High Beta Band  (25-40 Hz) | Gamma Band  (60-90 Hz) |
| --- | --- | --- | --- |
| CTL | 0.0101 ± 0.0007 | 0.0032 ± 0.00005 | 0.0014 ± 0.00003 |
| TTX | 0.0073 ± 0.0001* | 0.0083 ± 0.0003* | 0.059 ± 0.0017* |
| 6-OHDA | 0.0074 ± 0.0001* | 0.0022 ± 0.000043*;** | 0.00097 ± 0.00003*;** |

NRT: CTL, control; thalamic reticular nucleus; TTX: tetrodotoxin; 6-OHDA: 6-hydroxydopamine

*p < 0.016 vs Control **p < 0.016 TTX vs 6-OHDA

Table 4A. Percentage of cortical band changes in acute and chronic DA depletion state in comparison to control

| Cortex | Delta Band  (2-4 Hz) | Theta Band  (4-8 Hz) | Alpha Band  (8-13 Hz) | Low Beta Band  (13-25 Hz) | High Beta Band  (25-40 Hz) | Gamma Band  (60-90 Hz) |
| --- | --- | --- | --- | --- | --- | --- |
| TTX | -40.7% | -32.3% | 3.9% | 34.9% | 62% | 1258.4% |
| 6-OHDA | -20.5% | -17.2% | -2.1% | 55.6% | 67.9% | 261.6% |

TTX: tetrodotoxin; 6-OHDA: 6-hydroxydopamine

Table 4B. Percentage of MTh band changes in acute and chronic DA depletion state in comparison to control

| MTh | Delta Band  (2-4 Hz) | Theta Band  (4-8 Hz) | Alpha Band  (8-13 Hz) | Low Beta Band  (13-25 Hz) | High Beta Band  (25-40 Hz) | Gamma Band  (60-90 Hz) |
| --- | --- | --- | --- | --- | --- | --- |
| TTX | -19.3% | -25.5% | -31.7% | -34.6% | -35.7% | -71.8% |
| 6-OHDA | -16.8% | -16.7% | -14.2% | 1.2% | 2.4% | -30.8% |

TTX: tetrodotoxin; 6-OHDA: 6-hydroxydopamine

Table 4C. Percentage of NRT band changes in acute and chronic DA depletion state in comparison to control

| NRT | Delta Band  (2-4 Hz) | Theta Band  (4-8 Hz) | Alpha Band  (8-13 Hz) | Low Beta Band  (13-25 Hz) | High Beta Band  (25-40 Hz) | Gamma Band  (60-90 Hz) |
| --- | --- | --- | --- | --- | --- | --- |
| TTX | -98.8% | -94.3% | -83.4% | -27.8% | 154.8% | 4055.1% |
| 6-OHDA | -49.4% | -53% | -51.4% | -27.2% | -32.1% | -31.6% |

TTX: tetrodotoxin; 6-OHDA: 6-hydroxydopamine
